# Vascular Immune Remodeling: A CD4 ^+^ T Cell–Driven Immune Trajectory Associated With Arterial Stiffness

**DOI:** 10.1111/acel.70568

**Published:** 2026-06-04

**Authors:** Yu Miao, Bangwei Chen, Changqing Zeng, Xiaofen Wu, Minglei Yang, Cuntai Zhang, Jianguo Zhang, Yutao Du, Tao Li, Lei Ruan

**Affiliations:** ^1^ Institute of Biomedical Research, Henan Academy of Sciences Zhengzhou China; ^2^ BGI Genomics Shenzhen China; ^3^ Department of Geriatrics, Tongji Hospital, Tongji Medical College Huazhong University of Science and Technology Wuhan China; ^4^ Department of Pathology The First Affiliated Hospital of Zhengzhou University Zhengzhou Henan China; ^5^ School of Public Health Hebei Medical University Shijiazhuang China; ^6^ School of Basic Medicine Hebei Medical University Shijiazhuang Hebei Province China

**Keywords:** arterial stiffness, CD4^+^ T cell dysfunction, chronic immune activation, immune remodeling, machine learning, TCRβ repertoire, vascular aging

## Abstract

Arterial stiffness is a hallmark of vascular aging and a major risk factor for cardiovascular disease. While immune aging—typically characterized by reduced T cell receptor (TCR) diversity and CD8^+^ memory expansion—contributes to this process, growing evidence suggests that peripheral immune dysregulation may impair vascular function through mechanisms distinct from classical immunosenescence. To investigate this, we profiled peripheral TCRβ repertoires from 563 adults without clinically overt cardiovascular disease and assessed their association with pulse wave velocity (PWV), a gold‐standard marker of arterial stiffness. Surprisingly, individuals with elevated PWV exhibited a paradoxical increase in TCR diversity, driven by broad remodeling of numerous low‐frequency CD4^+^ clonotypes that preferentially mapped to a naive‐like/early‐differentiation CD4^+^ compartment. Single‐cell transcriptomic analyses further indicated chronic activation, proliferative restraint, and dysfunctional immune states in these PWV‐associated CD4^+^ T cells. Furthermore, a machine learning model trained on these PWV‐associated repertoire features accurately identified individuals with high PWV (AUC = 0.817), suggesting potential utility in subclinical vascular risk stratification. These findings define a distinct, CD4^+^ T cell–driven immune remodeling process—termed “vascular immune remodeling”—that is dissociable from classical immunosenescence and may contribute to arterial stiffening, uncovering an unexpected immune trajectory in early vascular aging and offering new avenues for immune‐based vascular risk assessment and intervention.

## Graphical abstract

This study integrated arterial stiffness with peripheral TCRβ repertoire and single‐cell transcriptomics in adults without cardiovascular disease, revealing a CD4^+^ T cell–driven “vascular immune remodeling” trajectory distinct from classical immunosenescence: increased TCR diversity, broad expansion of low‐frequency public clones and virus‐associated clones, yet accompanied by chronic activation and functional impairment.

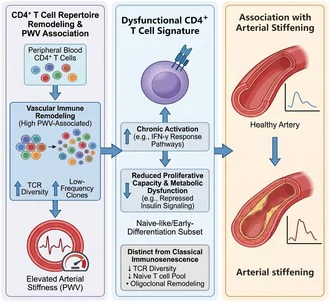

## 1 Introduction

Vascular aging, characterized by increased arterial stiffness and endothelial dysfunction, is a major determinant of cardiovascular disease (CVD) and late‐life mortality (Donato et al. 2018; Maier et al. 2023). As individuals age, arterial elasticity and structural integrity decline, increasing the risk of hypertension, atherosclerosis, and vascular calcification (Harvey et al. 2015; Sutton et al. 2023). Pulse wave velocity (PWV), the gold‐standard non‐invasive measure of arterial stiffness, serves as a widely accepted biomarker of vascular aging (Laurent et al. 2006; Marshall et al. 2024; Lu et al. 2023; Steppan et al. 2011; Fortier et al. 2017). While chronological aging has traditionally been regarded as the dominant driver of arterial stiffening, growing evidence indicates that the immune system is actively involved in, yet remains insufficiently understood in, the pathogenesis of this process.

Aging is often accompanied by chronic low‐grade inflammation, or inflammaging, which has been implicated in the pathogenesis of a wide range of age‐associated diseases, including CVD (Bektas et al. 2018; Santos‐Moreno et al. 2021). The classical model of immune aging, or immunosenescence, is defined by thymic involution, contraction of the naïve T cell pool, reduced T cell receptor (TCR) diversity, and expansion of antigen‐experienced T cells, often resulting in dysfunctional immune profiles (van de Sandt et al. 2023; Wang et al. 2025). These immune changes are believed to compromise immunosurveillance and promote chronic vascular inflammation. While prior studies have linked immunosenescence to vascular stiffness (Singh et al. 2024), it remains unclear whether additional forms of immune dysregulation, distinct from classical immunosenescence, may also contribute to early vascular dysfunction.

T cells are central to adaptive immunity, recognizing antigens through highly diverse TCRs generated by stochastic recombination of V(D)J gene segments (Laydon et al. 2015). The complementarity‐determining region 3 (CDR3) of the TCR is highly variable and governs antigen specificity (Rosati et al. 2017). High‐throughput sequencing of the TCRβ chain has enabled detailed profiling of peripheral immune repertoires, providing insights into immune competence, clonal architecture, and disease‐related immune states (Woodsworth et al. 2013; Robins 2013). As such, the TCRβ repertoire offers a valuable lens through which to explore systemic immune remodeling and its implications for vascular health.

Classical models of immune aging emphasize a progressive decline in TCR diversity, with clonal skewing and oligoclonal expansion of memory T cells driven by chronic infections or antigenic stimulation (van de Sandt et al. 2023; Mittelbrunn and Kroemer 2021; Britanova et al. 2016; Weng 2023; Gil et al. 2015). This model predicts a narrowed immune repertoire in aging individuals (Briceño et al. 2016). However, emerging data suggest that under certain inflammatory conditions, the immune system may undergo a “non‐classical” remodeling process—marked by increased TCR diversity and accumulation of dysfunctional but persistently activated T cell clones (Hanson et al. 2020; Frimpong et al. 2022; Smithey et al. 2018; Guo et al. 2020). These findings challenge traditional views of immunosenescence and raise the possibility that repertoire expansion may sometimes reflect a dysregulated immune state rather than immune vigor. Whether this non‐classical remodeling occurs in the context of vascular dysfunction remains unknown.

In this study, we systematically investigated the relationship between peripheral immune repertoire remodeling and arterial stiffness (measured by PWV). By leveraging high‐throughput TCRβ repertoire sequencing in a large cohort of 563 adults free of clinically overt cardiovascular disease, we sought to identify immune signatures associated with PWV. We discovered a paradoxical increase in TCR diversity in individuals with elevated PWV, a finding distinct from classical immunosenescence. This phenomenon, which we term “vascular immune remodeling,” was predominantly driven by the expansion of numerous low‐frequency, dysfunctional CD4^+^ T cell clonotypes. In addition, we demonstrate that these TCR repertoire features can be integrated into a machine learning model to accurately identify individuals with subclinical vascular dysfunction, establishing a new framework for immune‐based cardiovascular risk assessment. Our findings provide a new framework for understanding the interplay between the immune system and vascular health and lay the groundwork for immune‐driven strategies in cardiovascular risk prediction and intervention.

## 2 Methods

### 2.1 Study Design

This study was conducted within the prospective Tongji‐BGI Research Cardiovascular Health cohort. Participants were recruited from adults undergoing physical examinations at Tongji Hospital, Wuhan, China, between July 2020 and May 2022. The inclusion criteria were as follows: (1) age ≥ 20 years; (2) no history of coronary heart disease, stroke, congenital heart disease, rheumatic heart disease, or pacemaker implantation; (3) no history of tumors, liver failure, or renal failure; and (4) no history of autoimmune disease or recent infectious disease.

Participants who did not meet these criteria were excluded. All participants provided written informed consent, and the study protocol was approved by the Ethics Committee of Tongji Medical College, Huazhong University of Science and Technology (Approval No. 2020S146).

For TCR repertoire sequencing, 600 samples were initially prepared to accommodate potential technical loss. Among them, 574 samples passed the predefined DNA quality requirements for sequencing (DNA concentration > 67 ng/μL and total DNA amount ≥ 1.2 μg), whereas the remaining 26 samples were excluded because of insufficient DNA quantity or concentration. Thus, this source of missingness was nonrandom and was determined by objective laboratory quality‐control criteria rather than participant availability.

Among the 574 sequenced samples, 11 were further excluded from downstream analyses requiring integrated clinical covariates because most of their clinical information was missing. Accordingly, the final analytic cohort for downstream clinical‐integrative analyses comprised 563 participants (TORCH).

### 2.2 Definitions of Outcomes, Exposures, Predictors, and Confounders

The primary outcome was arterial stiffness, assessed as baPWV (cm/s). For each participant, PWV_Mean was calculated as the average of PWV_Rba and PWV_Lba and was used in downstream statistical and classification analyses. The main exposures included TCRβ repertoire characteristics, such as unique clone counts and diversity indices. Predictors and potential confounders comprised demographic variables (age, sex), laboratory parameters (systolic and diastolic blood pressure, pulse, and body mass index, uric acid, triglycerides, total cholesterol, erythrocyte pressure volume, hemoglobin, glucose, urine pH, glycated hemoglobin, mean hemoglobin concentration, creatinine, urea, bilirubin, homocysteine, and urine specific gravity). These variables were selected based on clinical relevance to vascular function and immune status.

### 2.3 Data Sources and Assessment Methods

Demographic information and clinical variables were collected at study visits through structured interviews and standardized laboratory assessments. Left and right brachial‐ankle pulse wave velocity (PWV_Lba and PWV_Rba) were measured in the supine position after a 10‐min rest using an automatic waveform analyzer operated by trained technicians. Two measurements were obtained on each side according to the standardized protocol. For downstream analyses, PWV_Mean was calculated for each participant as the average of the final PWV_Rba and PWV_Lba values. Peripheral blood samples were collected, and genomic DNA was isolated for amplification of the TCRβ complementarity‐determining region 3 (CDR3) using multiplex PCR, followed by high‐throughput sequencing. Laboratory variables were measured using standardized clinical chemistry methods. All measurements were performed consistently across participants.

### 2.4 Measurement of baPWV

Left and right brachial‐ankle pulse wave velocity (PWV_Lba and PWV_Rba) were measured using an automatic waveform analyzer operated by trained technicians. Participants were positioned supine and had electrocardiogram electrodes placed on their wrists, along with cuffs fitted around their upper arms and ankles. Following a 10‐min rest period to ensure physiological stability, the pulse waveforms were recorded using a semiconductor pressure sensor. Two measurements were obtained on each side according to the standardized protocol, and the results were reported in centimeters per second (cm/s). For downstream analyses, PWV_Mean was calculated for each participant as the average of the final PWV_Rba and PWV_Lba values.

### 2.5 Assessment of Inflammation Level

To evaluate the systemic inflammatory status of the study participants, we calculated inflammatory indices Systemic Immune‐Inflammation Index (SII) using the number of different types of cells in the peripheral blood (Mangalesh et al. 2024; Tanacan et al. 2023).

$$\mathrm{SII}=\text{Platelet Count}\times \frac{\text{Neutrophil Count}}{\text{Lymphocyte Count}}$$

SII indices are used to assess the level of inflammation of the sample. Higher values of these indices indicate elevated systemic inflammation, which was hypothesized to be associated with vascular aging.

### 2.6 Data Preprocessing and Imputation

Clinical data were meticulously preprocessed to ensure robustness and reliability for subsequent regression analyses. Initially, samples with more than 80% missing values were excluded to eliminate incomplete records. Following this, variables exhibiting over 80% missing data or a high frequency of a single value were removed to discard unreliable or non‐informative features. The remaining missing values were addressed using the missForest (version 1.5) algorithm, which employs a random forest‐based imputation method to accurately estimate and fill in missing data points. After imputation, the dataset was standardized to scale the variables, facilitating fair comparisons and enhancing the performance of regression models.

### 2.7 Single and Multiple Variable Regression Analyses

Subsequent regression analysis encompassed both univariate and multivariate approaches to identify significant predictors of the response variable, PWV_Mean. In the univariate analysis, each predictor was individually regressed against PWV_Mean to assess its association, with variables achieving a *p*‐value below 0.05 being selected for multivariate modeling. To mitigate multicollinearity, variance inflation factors (VIF) were calculated, and predictors with VIF values exceeding 10 were excluded from the multivariate analysis. A stepwise selection process based on the Akaike Information Criterion (AIC) was then employed to determine the optimal set of predictors for the final regression model. The final regression model was visualized using forest plots, highlighting the Estimates and significance levels of the selected predictors.

### 2.8 TCRβ Repertoire Sequencing

For each participant, 3 mL of peripheral blood was collected in EDTA‐anticoagulated tubes. Genomic DNA was extracted using the AxyPrep Blood Genomic DNA Mini Kit and stored at −80°C. TCRβ repertoire libraries were prepared from genomic DNA using a multiplex PCR strategy adapted from our previously established amplification framework, in which pooled forward primers targeting TRBV gene segments and pooled reverse primers targeting TRBJ gene segments were used to amplify the rearranged TCRβ CDR3 region (Liu et al. 2019). Briefly, 1.2 μg of input DNA was used for each sample. After purification, PCR products were used for library construction and subjected to 150‐bp paired‐end sequencing on the DNBSEQ‐T1 platform.

### 2.9 Data Processing

Raw FASTQ reads were preprocessed using fastp (version 0.20.1) prior to downstream analysis. Bases with a Phred quality score < 20 were considered low quality, and reads containing > 10% low‐quality bases or > 5 ambiguous bases (N) were discarded. In addition, the first 3 bases of both read 1 and read 2 were trimmed. The cleaned reads were then aligned to the IMGT database. CDR3 sequences, together with V, D, and J gene assignments, were identified and quantified using MiXCR (version 4.5.0) with default parameters (Bolotin et al. 2015). Only sequences with valid V and J assignments and productive CDR3 rearrangements were retained for downstream analyses. Non‐productive sequences, including those containing stop codons, pseudogene assignments, or CDR3 nucleotide sequences not divisible by three, were excluded. In addition, CDR3 sequences shorter than three amino acids or lacking the canonical cysteine at the start and phenylalanine at the end were filtered out.

### 2.10 Propensity Score Matching and Comparative Analysis of TCRβ Repertoire Characteristics

We performed propensity score matching (PSM) to reduce confounding between the PWV_High and PWV_Low groups. For comparative analyses, participants were first dichotomized according to PWV_Mean, with PWV_High defined as ≥ 1300 cm/s and PWV_Low defined as < 1300 cm/s. PSM was then conducted using the nearest‐neighbor method with a caliper width of 0.1 via the MatchIt R package (version 4.7.0) (Ho et al. 2007), with a 1:1 matching ratio. This procedure yielded a PSM‐matched development set of 71 matched pairs. The remaining 421 participants were retained as the internal validation set for subsequent model evaluation. The quality of matching was evaluated and visualized using love plots generated by the cobalt package (version 4.5.4), confirming adequate covariate balance between the matched groups. Subsequent comparative analyses focused on evaluating TCRβ repertoire characteristics between the matched PWV_High and PWV_Low groups. Throughout the manuscript, “TORCH cohort” refers to the full recruited study population (*n* = 563), “PSM‐matched development set” refers to the matched samples used for comparative analyses and model development, and “internal validation set” refers to the remaining participants used for model evaluation.

### 2.11 Identification and Analysis of Public Clones

Public Clones were defined as identical CDR3 amino acid sequences present in more than 50% of samples within a given group, consistent with our previous repertoire‐based analysis framework (Fang et al. 2025). To control for differences in group size, random sampling was performed within each group to assess the effect of increasing sample size on the number of Public Clones.

### 2.12 Identification of PWV‐Related Repertoire Functional Units (RFUs)

To enable repertoire‐level comparison across individuals, TCR clonotypes were grouped into repertoire functional units (RFUs) using a previously published sequence‐similarity‐based RFU framework (Yu et al. 2024). In this framework, sequence‐similar TCRs are grouped into shared repertoire‐level units that can be quantified across samples.

PWV‐associated RFUs were identified using a two‐step procedure in the PSM‐matched development set (71 matched pairs). First, Spearman correlation analysis was performed between the abundance of each individual RFU and PWV_Mean, and RFUs meeting the predefined criteria (correlation coefficient > 0.2, raw *p*‐value < 0.05, and Benjamini–Hochberg adjusted *p*‐value < 0.25) were retained as candidate PWV‐associated RFUs, here termed PWV_related_All_RFUs. Second, both RFU abundance and PWV_Mean were adjusted for the covariates independently associated with PWV in the multivariable regression model, including age, BMI, pulse, urine pH, SBP, DBP, and sex. Residuals from these models were then used for correlation analysis, and RFUs with residual‐based correlation coefficient > 0.1 and FDR < 0.05 were defined as PWV_related_RFUs.

### 2.13 TCRβ Repertoire Characteristics and Vascular Stiffness

We constructed predictive regression models (partial least squares regression) and classification models (LASSO regression) to investigate the relationship between TCRβ repertoire characteristics—such as unique clone numbers and immune diversity indices—and vascular stiffness. Ten‐fold cross‐validation was performed within the PSM‐matched development set (71 matched pairs), and model performance was subsequently evaluated in the internal validation set comprising the remaining 421 participants. The accuracy of the regression models was evaluated using RMSE and R‐squared, while the classification models were assessed by the area under the ROC curve (AUC).

### 2.14 CDR3 Sequence Similarity Analysis

CDR3 sequence similarity was evaluated using complementary sequence‐level and graph‐based approaches. TCR CDR3 amino acid sequences were clustered using the clustringr package (version 1.0; available at https://github.com/dan‐reznik/clustringr), with sequence neighborhoods defined on the basis of Levenshtein distance (maximum distance = 2) (Miao et al. 2023). For quantitative assessment, sequence similarity was summarized using normalized Levenshtein distance and BLOSUM62‐based pairwise similarity scores. Specifically, Levenshtein distances were normalized by the maximum length of each sequence pair, and BLOSUM62‐based similarities were calculated from global pairwise alignments and scaled by the geometric mean of the corresponding self‐alignment scores. To account for differences in the numbers of clonotypes between groups, bootstrap resampling was performed for balanced comparisons where indicated. Clustering topology and sequence neighborhoods were visualized using the ggplot2 package (version 3.5.1).

### 2.15 Pathological Clone Annotation in the TCRβ Repertoire

Custom Perl scripts were used to annotate TCRβ CDR3 sequences, leveraging the PIRD (Zhang et al. 2020), VDJdb (Shugay et al. 2018), and McPAS (Tickotsky et al. 2017) databases. Disease‐associated clones were categorized into four groups: cancer, autoimmune diseases, pathogen‐related diseases, and others. For each analysis set, the proportion of pathological clonotypes was calculated as the number of unique CDR3 amino acid sequences assigned to a given category divided by the total number of unique CDR3 amino acid sequences analyzed (Miao et al. 2023).

### 2.16 Single‐Cell Clustering and Marker‐Based Annotation

Single‐cell RNA‐seq and paired TCR‐seq data were obtained from a previously published healthy single‐cell dataset derived from GEO (GSE157007) (Luo et al. 2022). Although the original resource also included cord blood and frail older adult samples, the present study included only the healthy Young and Old samples, comprising nine donors in total (three young adults and six older adults) and 56,641 cells after subsetting and preprocessing. These data were used for cell‐type annotation and validation of the cellular origins of PWV‐related RFUs. Data were processed using the Seurat R package (version 4.4.0). Data were integrated and corrected for batch effects using the Harmony algorithm (version 1.0), applied to the top 20 principal components. Dimensionality reduction was conducted using t‐distributed stochastic neighbor embedding (t‐SNE), and cells were clustered using a shared nearest neighbor (SNN)‐based method with a resolution of 1.2. Clusters were annotated based on canonical marker genes identified using the FindAllMarkers function. Cell types were assigned based on known lineage markers.

### 2.17 Mapping of PWV‐Associated Repertoire Features to the Single‐Cell Reference Atlas

To investigate the cellular context of PWV‐associated repertoire features, we mapped PWV_related_RFUs to a publicly available healthy single‐cell reference dataset with paired scRNA‐seq and scTCR‐seq data (GSE157007), in which T‐cell states had been annotated on the basis of transcriptomic profiles. Reference cells carrying TCRs assigned to PWV_related_RFUs were identified, and their distribution across major T‐cell lineages and annotated CD4^+^ T‐cell subsets was quantified.

To further refine the CD4^+^ cellular context of PWV‐associated clonotypes, we performed exact‐match mapping of the 114 TCRβ clonotypes significantly enriched in the PWV_High group to the same paired single‐cell TCR reference atlas at the CDR3 amino acid sequence level. Matched clonotypes were assigned to the annotated cell states of the corresponding reference cells, and their distribution across CD4^+^ T‐cell subsets was summarized. Because the bulk TCRβ repertoire data and the public single‐cell reference atlas were derived from independent cohorts rather than matched individuals, these analyses were used to infer reference‐atlas‐based lineage/state correspondence rather than direct phenotypic identity.

### 2.18 Differential Gene Expression and Enrichment Analysis

We performed differential expression analysis between RFU^+^ T cells and their corresponding lineage background using Seurat's FindMarkers function. Significant genes were defined as those with adjusted *p*‐values < 0.05 and absolute log2 fold change > 0.25.

Gene Set Enrichment Analysis (GSEA) was conducted using the clusterProfiler package (version 4.14.4). Enrichment was performed against MSigDB Hallmark and KEGG pathway gene sets. Enrichment scores were visualized using the enrichplot and ggplot2 packages. Pathways with |NES| > 1 and software‐reported adjusted *p*‐values (*p*.adjust) < 0.05 were considered significantly enriched.

### 2.19 Feature Selection and Classification Model Construction

Feature selection was performed using LASSO regression (glmnet, version 4.1.8) and recursive feature elimination (RFE) with random forest ranking (caret, version 7.0.1). For LASSO, variables with coefficients greater than zero at the optimal lambda value, identified via 10‐fold cross‐validation, were retained. RFE was conducted using bootstrap resampling with 10 iterations, employing random forest to rank feature importance. Features with positive importance scores were selected. The features selected by both methods were then used for subsequent modeling.

Thirteen supervised machine learning algorithms were implemented to classify arterial stiffness status (PWV_High vs. PWV_Low), including logistic regression, naive Bayes, k‐nearest neighbors, support vector machines (linear and radial kernels), neural networks, random forest, decision trees, gradient boosting (GBM, XGBoost, LightGBM, CatBoost), LASSO classifier, and AdaBoost. All models were developed using five‐fold cross‐validation in the PSM‐matched development set and subsequently evaluated in the internal validation set. ROC curves and AUCs were computed using the pROC package (version 1.18.5).

### 2.20 Precision‐Recall Curve Analysis

To address potential class imbalance in the internal validation set, precision‐recall (PR) curves were computed using the pROC R package. For the best‐performing model (Naïve Bayes using RFE‐selected features), PR‐AUC was calculated, and baseline precision was indicated.

### 2.21 Statistical Analysis

Non‐paired sample comparisons were performed using the Wilcoxon rank‐sum test, with data visualizations produced using R version 4.3.1. Univariate analyses were used as an initial screening step, and raw *p*‐values < 0.05 were used to retain potentially relevant variables for multivariable modeling. Univariate analyses were used as an initial screening step, and raw *p*‐values < 0.05 were used to retain potentially relevant variables for multivariable modeling. Multiple‐testing correction was performed using the Benjamini–Hochberg procedure to control the false discovery rate (FDR) for analyses in which adjusted *p*‐values were specifically reported, unless adjusted *p*‐values were provided directly by the analysis software.

## 3 Results

### 3.1 Age, Sex, and Blood Pressure as Dominant Determinants of Pulse Wave Velocity in Healthy Individuals

**FIGURE 1:**
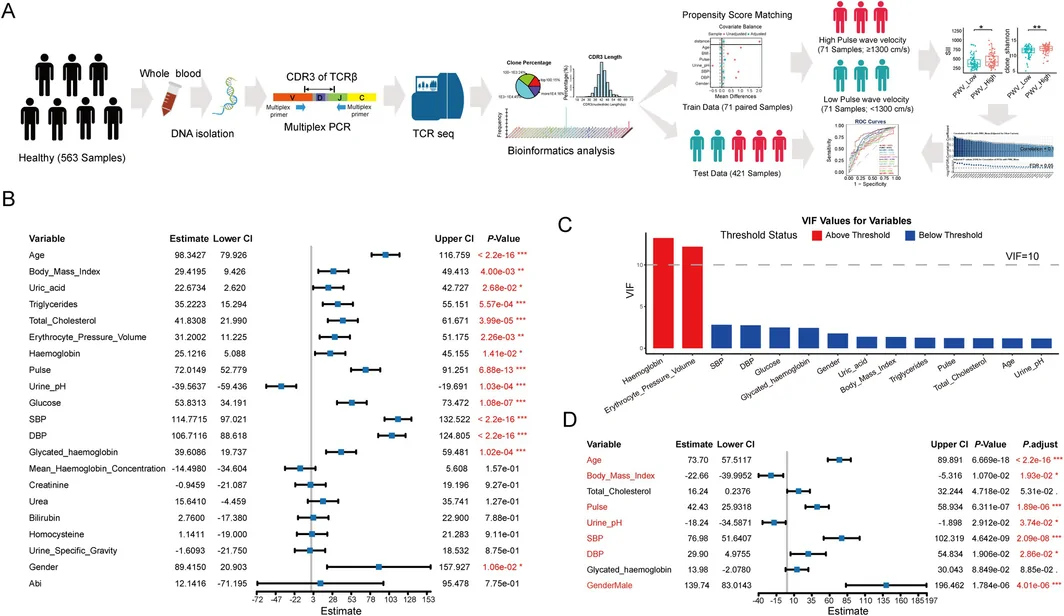
Physiological and clinical determinants of arterial stiffness in healthy individuals. (A) Schematic overview of the study design. PWV was measured in 563 healthy individuals. TCRβ sequencing was performed on peripheral blood samples. A matched cohort of PWV_High (≥ 1300 cm/s) and PWV_Low (< 1300 cm/s) individuals was constructed using propensity score matching (PSM). (B) Forest plot showing univariate linear regression results for physiological variables associated with PWV. Variables shown in red font are significantly associated (*p* < 0.05). (C) Bar plot displaying the VIF values for selected clinical variables. Variables with VIF > 10 (shown as red bars) were excluded from the multivariate regression analysis. (D) Forest plot showing multivariate linear regression results for physiological variables associated with PWV. Variables shown in red font are significantly associated (*p*.adjust < 0.05). Significance is indicated as follows: *p.adj < 0.05; **p.adj < 0.01; ***p.adj < 0.001.

Arterial stiffness is a hallmark of vascular aging and can be non‐invasively assessed by measuring PWV. To explore potential immune mechanisms underlying arterial stiffening, we measured PWV and performed TCRβ sequencing on peripheral blood samples from the TORCH cohort of 563 individuals without clinically overt cardiovascular disease (Figure S1A and Table S1). The overall study design is summarized in Figure 1A. Genomic DNA was extracted from whole blood, followed by multiplex PCR amplification of the TCRβ CDR3 region, high‐throughput sequencing, and downstream bioinformatic analysis. To account for potential confounders, participants in the TORCH cohort were first dichotomized according to PWV_Mean using a prespecified cutoff of 1300 cm/s, with PWV_High defined as ≥ 1300 cm/s and PWV_Low defined as < 1300 cm/s. We then performed 1:1 propensity score matching between the two groups, yielding a PSM‐matched development set of 71 matched pairs with comparable distributions of physiological and metabolic variables (Figure S1C and Table S2). The remaining 421 participants were retained as the internal validation set for subsequent model evaluation.

We first conducted univariate linear regression to identify physiological and biochemical factors associated with PWV. Age, systolic and diastolic blood pressure (SBP and DBP), pulse rate, blood glucose, uric acid, erythrocyte pressure volume, hemoglobin, and several metabolic indicators—including BMI, total cholesterol, triglycerides, and glycated hemoglobin (HbA1c)—were all significantly associated with PWV in univariate analysis (*p*‐value < 0.05) (Figure 1B). Notably, age, blood pressure, and glycemic levels have been previously implicated in arterial stiffening and vascular aging (Baier et al. 2018; Shin et al. 2011).

To reduce multicollinearity and potential confounding, variables with high variance inflation factors (VIF > 10) were excluded (Figure 1C). A multivariate linear regression model was then constructed using stepwise regression. After false discovery rate (FDR) adjustment, we found age (Estimate = 73.7, 95% CI: 57.5–89.9, *p*.adj < 2.2 × 10^−16^), systolic blood pressure (Estimate = 77.0, 95% CI: 51.6–102.3, *p*.adj = 2.09 × 10^−8^), and male sex (Estimate = 139.7, 95% CI: 83.0–196.5, *p*.adj = 4.01 × 10^−6^) emerged still as the strongest independent determinants of PWV (*p*.adj < 0.05) (Figure 1D). Male was associated with substantially higher PWV compared to females reflecting established sex differences in vascular aging (Lu et al. 2023), which may be related to the role of estrogen in women's blood vessels (DuPont et al. 2019). Notably, BMI demonstrated a negative association with PWV in the multivariate model (Estimate = −22.7, 95% CI: −40.0 to −5.3, *p*.adj = 0.019), in contrast to its positive association in univariate analysis. This inverse trend aligns with findings in a Chinese health assessment cohort, where higher BMI was independently associated with lower arterial stiffness (Tang et al. 2020). This may reflect the buffering effect of subcutaneous fat against vascular damage under specific hemodynamic conditions. Having confirmed the influence of these classical drivers, we next sought to determine whether a distinct immunological signature of arterial stiffness exists after rigorously controlling for these confounding factors.

### 3.2 Arterial Stiffness Correlates With Enhanced TCR Diversity Driven by Expansion of Low‐Frequency Clones

To explore potential immunological underpinnings of arterial stiffness independent of classical physiological drivers, we utilized the PSM‐defined PWV_High and PWV_Low groups (Figure S1B,C), which were matched for age, sex, blood pressure, BMI, and other confounders (Figure S1D and Table S2). This matching allowed us to isolate immune‐related features potentially contributing to increased PWV.

**FIGURE 2:**
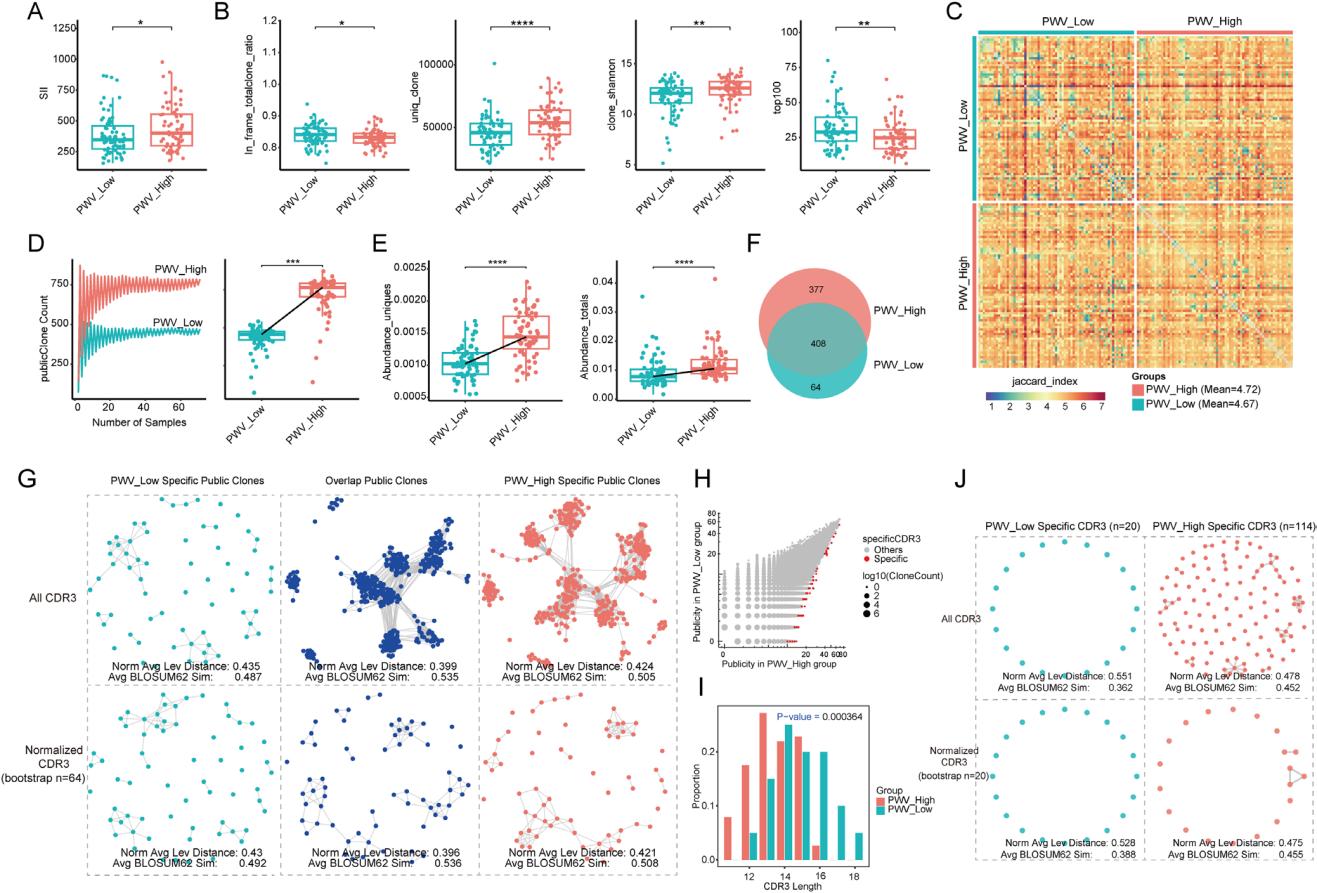
TCRβ repertoire features and clonal architecture in individuals with different PWV. (A) Boxplot showing systemic inflammation index across PWV_High and PWV_Low groups. (B) Boxplots showing TCRβ repertoire features, including in‐frame productive ratio, unique clone count, Shannon diversity index, and cumulative frequency of the top 100 most abundant clones. (C) Heatmap showing pairwise Jaccard similarity indices between TCRβ repertoires from individuals in PWV_High and PWV_Low groups. (D) The line plot (left) shows the change in the number of public clones in the two groups as the sample size increases; the boxplot (right) compares the difference in the overall number of public clones in each group. (E) The boxplot (left) shows the difference of unique public clones proportion, and the boxplot (right) shows the difference of total public clones abundance. (F) Venn diagram showing the number of public clonotypes specific to each group or shared between PWV_High and PWV_Low groups. (G) Network graphs showing sequence similarity among public CDR3 clonotypes. Top row: All CDR3s. Bottom row: Normalized CDR3s. Groups include PWV_Low‐specific, shared, and PWV_High‐specific clonotypes. (H) Scatterplot showing clone publicity (number of individuals sharing each clone) in PWV_Low and PWV_High groups. PWV‐associated clones identified by Fisher's exact test are highlighted. (I) Histogram comparing the CDR3 length distributions of clonotypes significantly enriched in the PWV_High group and clonotypes significantly enriched in the PWV_Low group. (J) Sequence similarity network graphs comparing clonotypes significantly enriched in the PWV_High group and clonotypes significantly enriched in the PWV_Low group. Significance is indicated as follows: *p < 0.05; **p < 0.01; ***p < 0.001; ****p < 0.0001.

Within this PSM‐matched development set, we observed that individuals with higher PWV exhibited significantly elevated systemic inflammation (Figure 2A), suggesting a potential correlation between vascular stiffening and immune activation. We then investigated the underlying TCR repertoire architecture. Strikingly, and in sharp contrast to the repertoire contraction typical of classical immunosenescence and most autoimmune diseases (Miao et al. 2023; Amoriello et al. 2021; Lanz et al. 2022), the PWV_High group exhibited significantly greater TCRβ repertoire diversity (measured by the Shannon index) (Figure 2B). Conversely, the cumulative frequency of the top 100 most abundant clones was significantly lower in the PWV_High group (Figure 2B). These findings indicate a positive, independent association between PWV and increased TCRβ repertoire diversity. This association persisted after adjusting for covariates across the full cohort (Figure S2A). Intriguingly, we also noted a significantly lower proportion of in‐frame TCRβ clonotypes within the PWV_High group (Figure 2B), suggesting an accumulation of potentially non‐functional clones with increased arterial stiffness. This finding bears resemblance to the age‐associated increase in translational errors observed in various tissues (Böttger et al. 2025). Furthermore, significant differences were observed in the utilization frequencies of V genes (Figure S2B), J genes (Figure S2C), and VJ pairings (Figure S2D), as well as in CDR3 sequence lengths, with the PWV_High group displaying significantly shorter CDR3 lengths overall (Figure S2E).

Next, we assessed clonal similarity between samples within each group. The PWV_High group exhibited higher Jaccard similarity scores than the PWV_Low group (Figure 2C), prompting us to investigate the presence and characteristics of public clonotypes (shared across multiple individuals). The PWV_High group exhibited a significantly greater number and proportion of public clonotypes, which also displayed markedly higher cumulative abundance within the overall TCR repertoire (Figure 2D,E). Previous studies have suggested that shared public clones among individuals with similar traits may reflect common immune exposures or responses (van de Sandt et al. 2023; Foth et al. 2020; Warren et al. 2011). This expansion of low‐frequency public T cell clones in the PWV_High group likely reflects immune system activation in response to shared antigenic exposures.

Further characterization of these public clonotypes revealed distinct sequence features among clones specific to the PWV_High group, specific to the PWV_Low group, or shared between both groups (Figure 2F,G). We quantified sequence similarity using the mean Levenshtein distance; similarity was lowest among PWV_Low‐specific public clones, intermediate for PWV_High‐specific clones, and highest for clones shared by both groups. These differences persisted even after controlling for the number of public clones via random sampling (Figure 2G).

Finally, to identify specific TCR clonotypes associated with PWV status, we performed Fisher's exact test on all clones across the matched groups. This identified 114 TCR clonotypes significantly enriched in the PWV_High group and 20 clonotypes significantly enriched in the PWV_Low group (Figure 2H). Compared with PWV_Low‐enriched clonotypes, PWV_High‐associated clonotypes exhibited shorter CDR3 lengths and higher sequence similarity (Figure 2I,J). Similar trends were also observed in comparisons with randomly sampled control repertoires (Figure S3A,B).

Subsequently, we conducted pathology‐associated clonotype annotation of the TCR clonotypes significantly enriched in the PWV_High and PWV_Low groups. Pathogen‐related annotations were markedly more frequent among PWV_High‐enriched clonotypes than among PWV_Low‐enriched clonotypes (67/114, 58.8% vs. 3/20, 15.0%; Fisher's exact test, *p* < 0.001; Figure S3C,D). Consistently, a randomly sampled control repertoire also showed a much lower annotation rate (67/114, 58.8% vs. 2/114, 1.8%; Fisher's exact test, *p* < 0.001; Figure S3E). Further stratification showed that the annotated PWV_High‐associated clonotypes were predominantly linked to common viral infections, particularly CMV and influenza (Figure S3F). Although these sequences were mainly virus‐associated, their diffuse, low‐frequency distribution and the paradoxical increase in overall repertoire diversity differ from the oligoclonal contraction seen in classical memory responses. These atypical repertoire features prompted us to further resolve the cellular identity and functional states of these PWV‐associated clones at the single‐cell level.

### 3.3 Separate T Cell Dynamics Drive Immune Changes in Vascular Stiffness Versus Aging

Previous studies have established a strong correlation between increased arterial stiffness and chronological aging, as the progressive loss of arterial elasticity with age inevitably drives vascular stiffening (Lu et al. 2023; Mikael et al. 2017). Analysis of the TORCH cohort confirmed this association (Figure 1D). A critical question is whether the immune remodeling associated with arterial stiffness simply mirrors chronological aging or instead represents a partially dissociable immunological process with distinct repertoire features. To address this, we applied the previously described repertoire functional unit (RFU) framework to quantify repertoire‐level T‐cell features associated with age and vascular stiffness (Yu et al. 2024).

**FIGURE 3:**
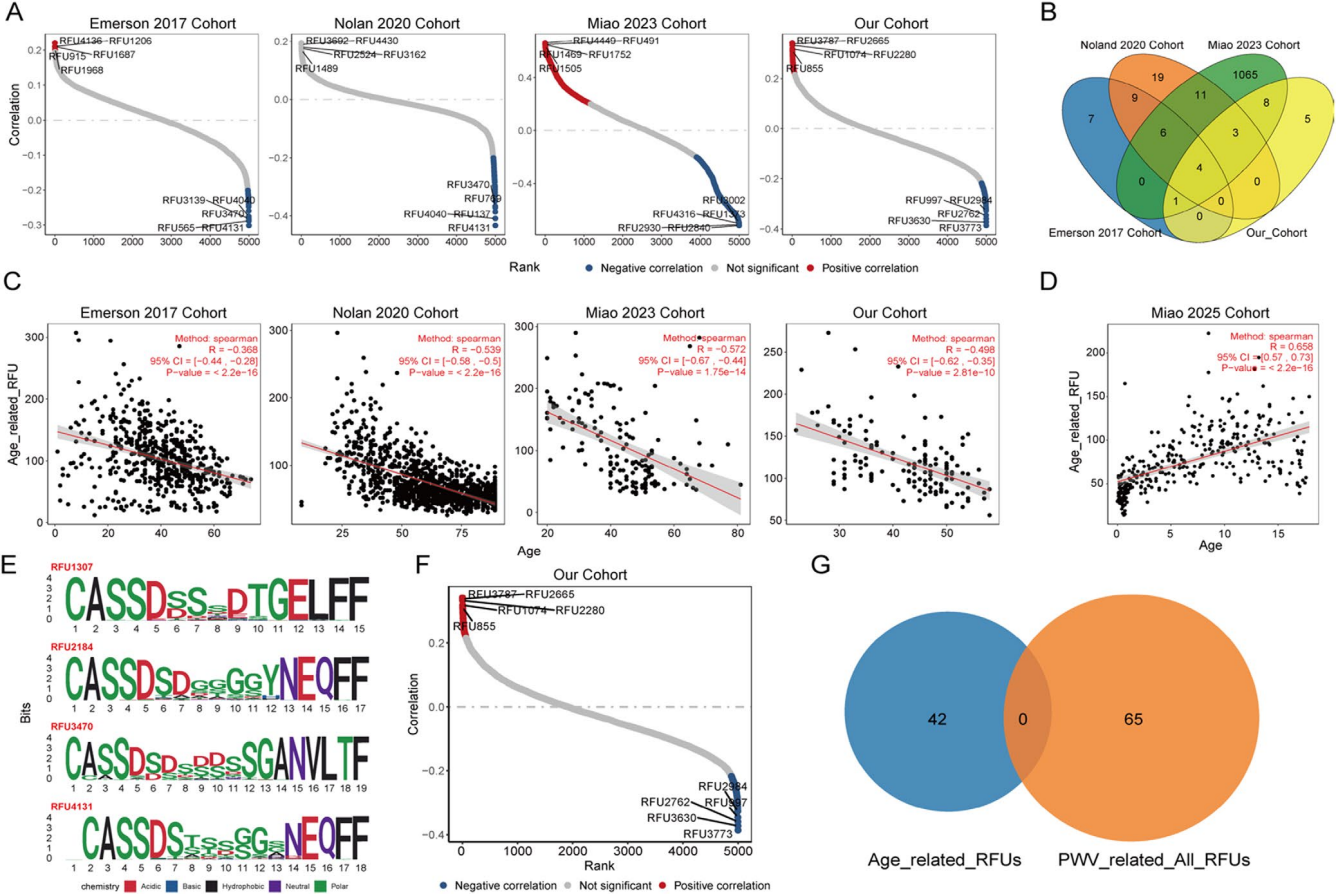
Comparative analysis of RFUs reveals independent T cell expansions in aging and arterial stiffness. (A) Correlation‐ranked plots showing age‐related RFUs in four adult cohorts. RFUs significantly correlated with age are marked in red (positive) and blue (negative). (B) Venn diagram showing the number of overlapping age‐related RFUs across the four cohorts. RFUs shared in at least two cohorts were defined as Age_related_RFUs. (C) Scatterplots showing negative correlations between Age_related_RFUs and chronological age in each adult cohort. Spearman correlation coefficients, 95% confidence intervals, and *p*‐values are indicated. (D) Scatterplot showing the correlation between Age_related_RFUs and age in our previously published study of a healthy pediatric cohort. (E) Representative CDR3 sequence motifs of four conserved Age_related_RFUs shared across multiple adult cohorts. (F) Correlation‐ranked plot showing PWV‐related RFUs (PWV_related_RFUs) identified from our cohort. Significant correlations with PWV are indicated. (G) Venn diagram showing the number of overlap RFUs between Age_related_RFUs and PWV_related_RFUs.

Using this framework, we defined a reference set of age‐related RFUs (Age_related_RFUs) across multiple adult TCR repertoire cohorts, including publicly available adult datasets and a healthy adult cohort from our previous study (Fang et al. 2025; Nolan et al. 2020; Emerson et al. 2017). RFUs were classified as Age_related_RFUs if they were significantly associated with chronological age and shared across at least two adult cohorts (Figure 3A,B). When examined in our previously published healthy pediatric cohort, these adult‐defined Age_related_RFUs increased progressively with age during childhood, whereas in adult populations, their abundance declined significantly with age (Figure 3C,D). This pattern across life stages is consistent with the published life‐stage trajectory of age‐associated RFUs, which rise after birth and subsequently decline during adult aging (Hu et al. 2024). Together, these findings suggest a dynamic, age‐phase‐dependent immune remodeling trajectory in which the same RFU signatures exhibit distinct patterns across different life stages. Importantly, these RFUs represent a quantifiable signature of immune aging at the TCR repertoire level, rather than a complete representation of immunosenescence. Representative sequence motifs from the most conserved Age_related_RFUs are shown in Figure 3E. These motifs exhibit conserved amino‐acid patterns at central CDR3 positions; for example, RFU1307 consistently presents Cysteine (C), Serine (S), Aspartic acid (D), and Threonine (T) at key anchor residues, which may reflect structural or antigen‐binding constraints.

Applying the same RFU framework to the PSM‐matched development set, we first identified 65 RFUs associated with PWV at the individual‐RFU level (PWV_related_All_RFUs; Figure 3F). Systematic comparison revealed a complete absence of overlap between PWV_related_All_RFUs and Age_related_RFUs (Figure 3G). This non‐overlap suggests that PWV‐associated repertoire features do not simply recapitulate published age‐associated RFU patterns, but instead may reflect an immunological remodeling process that is at least partially dissociable from canonical immunosenescence.

### 3.4 Expansion of CD4 ^+^ T Cell‐Derived Clonotypes Is Associated With Increased Arterial Stiffness

**FIGURE 4:**
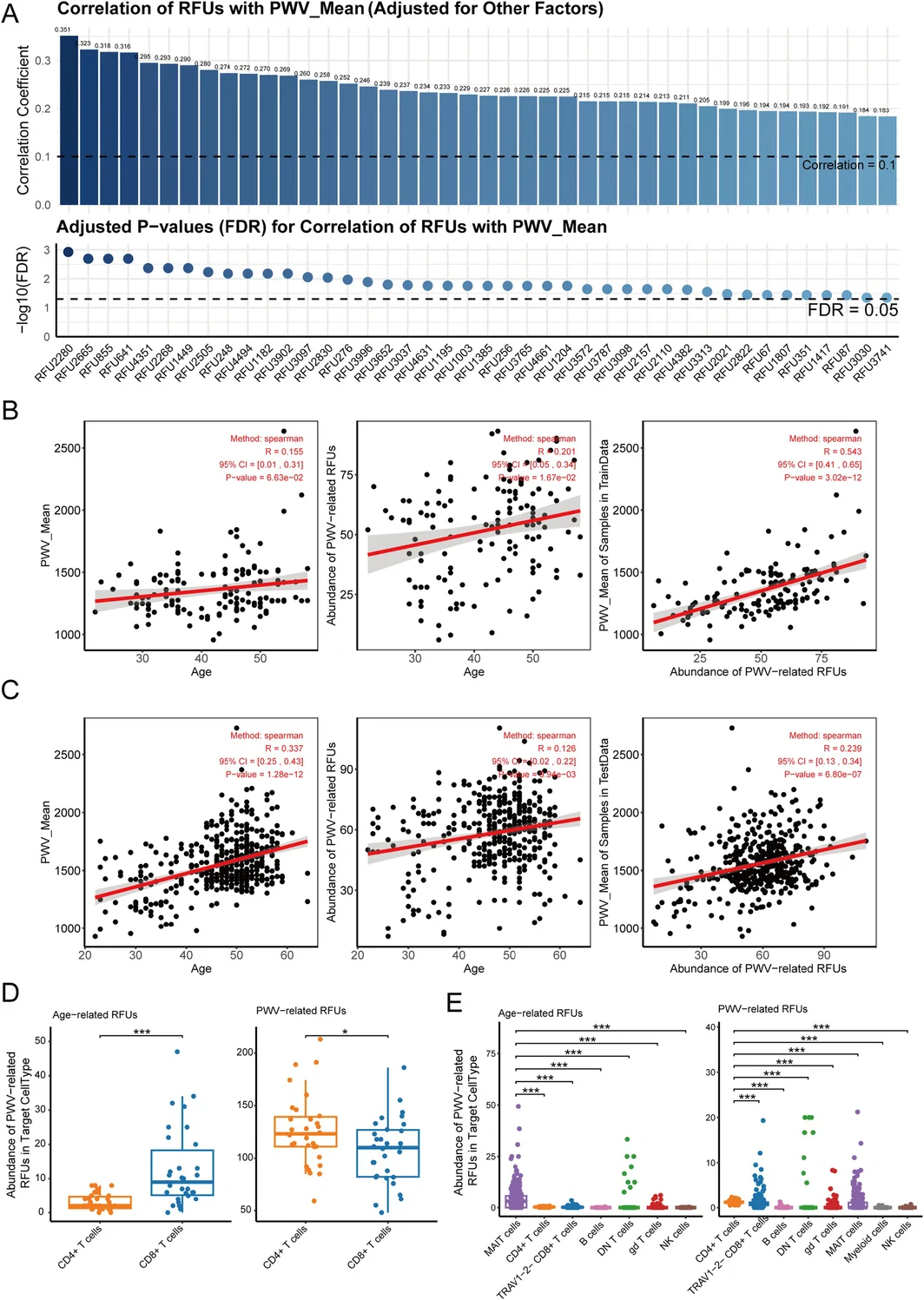
Identification and characterization of PWV‐associated RFUs and their cellular origins. (A) The correlation coefficients (Barplot) and corresponding adjusted *p*‐values (Scatterplot) between RFU abundance and mean PWV values after adjustment for physiological and biochemical covariates. Forty‐five RFUs were identified as independently associated with PWV (PWV_related_RFUs). FDR, false discovery rate. (B) Scatterplots from the training cohort (*n* = 71 pairs) showing: (left) correlation between PWV and age; (middle) correlation between age and abundance of PWV_related_RFUs; (right) correlation between PWV and abundance of PWV_related_RFUs. (C) Scatterplots from the test cohort (*n* = 421) showing the same relationships as in panel B. Correlation coefficients, 95% confidence intervals, and *p*‐values are shown in red. (D) Boxplots showing the relative abundance of Age_related_RFUs and PWV_related_RFUs within CD4^+^ and CD8^+^ T‐cell‐derived TCR repertoires. (E) Boxplots showing the abundance of Age_related_RFUs (left) and PWV_related_RFUs (right) across different immune cell subsets derived from single‐cell transcriptome and TCR sequencing data in a healthy cohort. Significance is indicated as follows: *p.adj < 0.05; ***p.adj < 0.001.

To further refine this PWV‐associated RFU set, we performed residual‐based covariate adjustment using the physiological and biochemical variables independently associated with PWV in the multivariable regression model (Figure 1D), including age, BMI, pulse, urine pH, SBP, DBP, and sex. Both RFU abundance and PWV were adjusted for these covariates, and residual‐based correlation analyses were then performed. This yielded 42 RFUs that remained significantly associated with PWV after covariate adjustment, which we defined as PWV_related_RFUs (Figure 4A).

We then conducted correlation analyses between age, PWV, and these RFUs in both the PSM‐matched development set (71 matched pairs) and the internal validation set (the remaining 421 participants). Although a direct correlation between age and PWV only showed a trend (*p*‐value = 0.06) in the PSM‐matched development set (Figure 4B, left), the overall data showed an increase in PWV with age (Figure 1D). In both the PSM‐matched development set and the internal validation set, we found the abundance of PWV_related_RFUs increased with age and was positively correlated with PWV (Figure 4B,C, middle and right). These findings indicate that PWV‐associated T‐cell remodeling continues to evolve with advancing age while remaining distinct from canonical age‐related TCR repertoire remodeling.

Given the observed non‐overlap between PWV_related_All_RFUs and age‐associated RFUs (Figure 3G), we hypothesized that these distinct RFU sets may originate from different T cell subsets. To verify this, we used a publicly available dataset of sorted CD4^+^ and CD8^+^ T‐cell repertoires to compare the abundance of Age_related_RFUs and PWV_related_RFUs between the two subsets. Surprisingly, Age_related_RFUs were significantly more abundant in TCRs from CD8^+^ T cells than from CD4^+^ T cells, whereas the opposite was true for PWV_related_RFUs (Figure 4D). These results indicate that Age_related_RFUs are predominantly derived from CD8^+^ T cells, whereas PWV_related_RFUs originate mainly from CD4^+^ T cells, suggesting distinct lineage involvement in age‐related versus vascular stiffness–related immune responses.

To validate these observations, we analyzed single‐cell transcriptomic and paired TCR‐seq data from a previously published public healthy single‐cell dataset. This confirmed that Age_related_RFUs were mainly produced by mucosal‐associated invariant T (MAIT) cell subsets, a finding consistent with recent literature (Wang et al. 2025; Hu et al. 2024), whereas PWV_related_RFUs were predominantly produced with CD4^+^ T‐cell subsets (Figure 4E). These results highlight distinct immunological mechanisms underlying age‐related and PWV‐related changes in the TCR repertoire, highlighting a predominantly CD4^+^ T cell‐centered immune remodeling pattern associated with arterial stiffness.

While RFU‐level mapping localized PWV‐associated repertoire signals predominantly to the CD4^+^ compartment, it did not reveal clear overrepresentation of specific canonical CD4^+^ subsets. We performed exact‐match mapping of the 114 clonotypes significantly enriched in the PWV_High group to the paired single‐cell TCR reference atlas and observed preferential representation within a naive‐like CD4^+^ compartment (Figure S4A). These analyses further refined the CD4^+^ cellular context of PWV‐associated repertoire features.

### 3.5 Chronic Immune Activation and Functional Impairment of CD4 ^+^ T Cells Underlie Elevated PWV

**FIGURE 5:**
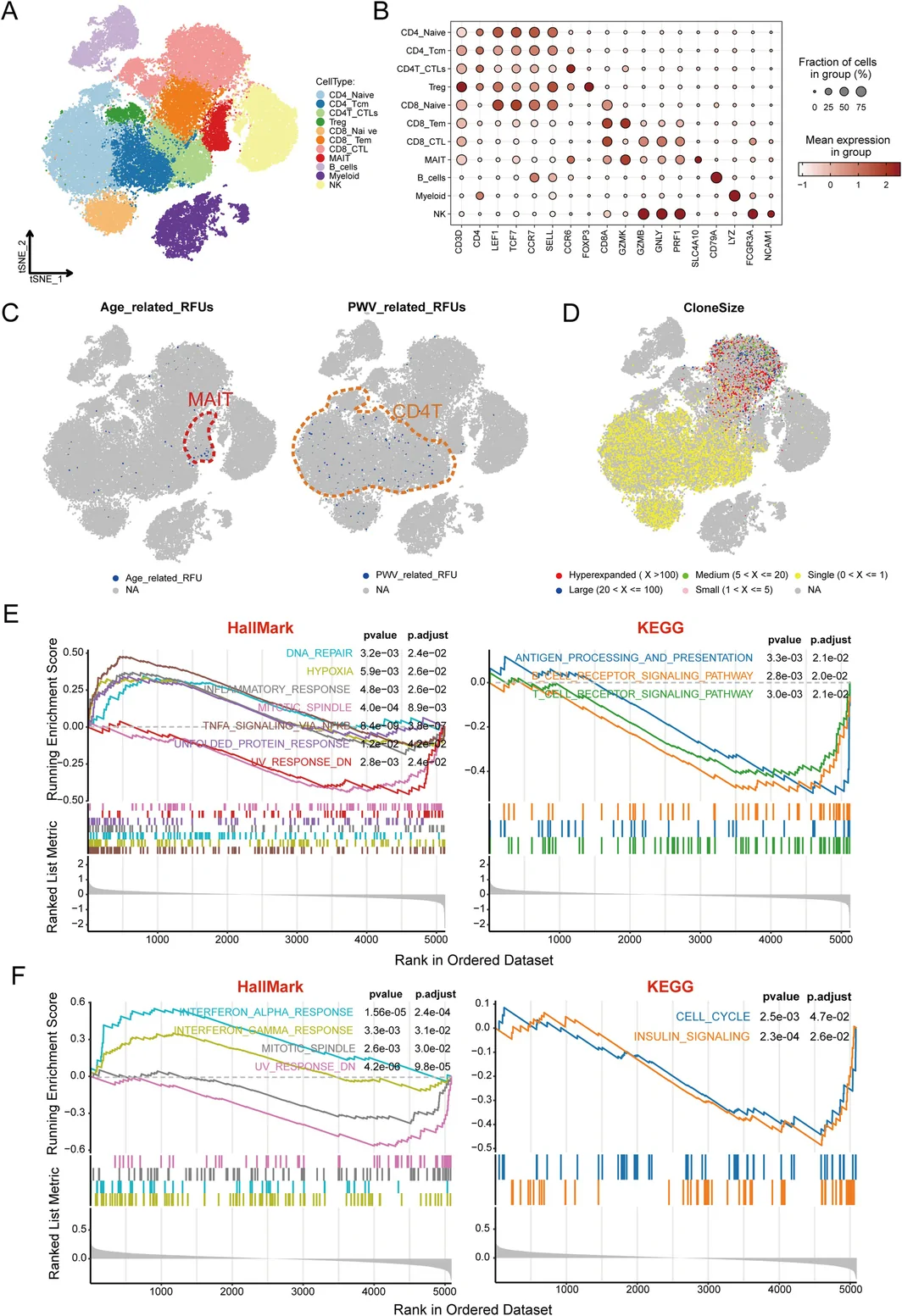
Single‐cell transcriptomic analysis identifies distinct expression programs in age‐ and PWV‐associated T cell subsets. (A) tSNE plot of single‐cell RNA‐seq data from healthy individuals showing 12 major immune cell subsets. (B) Dotplot showing the expression of representative marker genes across identified cell clusters. Dot size represents the proportion of cells expressing the gene; color indicates expression level. (C) Distribution of Age_related_RFUs and PWV_related_RFUs mapped to single‐cell clusters. Age‐related RFUs were enriched in MAIT cells; PWV‐related RFUs were enriched in CD4^+^ T cells. (D) Clonal expansion status of T cells visualized on tSNE plot. Cells are colored by clonal size (hyperexpanded, large, medium, or singletons). (E) Gene Set Enrichment Analysis result for Age_related_RFU^+^ MAIT cells compared to other MAIT cells. Left: Hallmark database. Right: KEGG database. (F) GSEA for PWV_related_RFU^+^ CD4^+^ T cells compared to other CD4^+^ cells. Left: Hallmark pathways. Left: Hallmark database. Right: KEGG database.

To further validate the cellular origins and transcriptional features of PWV‐related RFUs, we analyzed single‐cell RNA‐seq and paired TCR‐seq data from healthy young and older adults in a previously published dataset (Luo et al. 2022). Dimensionality reduction and clustering identified 12 major immune cell subsets (Figure 5A), with representative marker genes shown in Figure 5B.

Consistent with our previous TCR repertoire analysis result (Figure 4D,E), Age_related_RFUs were predominantly derived from MAIT subsets, whereas PWV_related_RFUs were enriched in CD4^+^ T cell subsets (Figure 5C, Figure S4B,C). Notably, both MAIT and CD4^+^ cells exhibited primarily monoclonal profiles, in contrast to CD8^+^ effector memory (Tem) and cytotoxic (CTL) subsets, which showed signs of substantial clonal expansion (Figure 5D).

Next, we performed differential analysis between Age_related_RFU^+^ MAIT cells and PWV_related_RFU^+^ CD4^+^ T cells to other cells of the same lineage (Table S3), followed by gene set enrichment analysis using the Hallmark and KEGG pathway databases. In age‐associated MAIT cells, pathways related to TNFα signaling via NF‐κB, DNA repair, hypoxia, inflammatory response, and mitotic spindle were significantly enriched, whereas pathways such as unfolded protein response, UV response down, antigen processing and presentation, B cell receptor signaling, and T cell receptor signaling were suppressed (Figure 5E). These findings indicate an aging‐related shift toward cellular stress and inflammation, coupled with reduced antigen responsiveness and signaling capacity—hallmarks of T cell immunosenescence (van de Sandt et al. 2023; Salminen et al. 2008; Zhao et al. 2023).

In contrast, PWV‐associated CD4^+^ T cells displayed upregulation of interferon‐α and interferon‐γ response, while cell cycle progression, UV response down, mitotic spindle, and insulin signaling pathways were repressed (Figure 5F). These transcriptional features suggest that CD4^+^ T cells in individuals with elevated PWV undergo chronic immune activation, accompanied by cell cycle arrest and impaired proliferative capacity. This phenotype mirrors T cell dysfunction and maladaptive immune responses observed in the pathogenesis of atherosclerosis and vascular aging, characterized by proliferation arrest and features of immune exhaustion driven by persistent inflammation (McLaren and Ramji 2009; Gaddis et al. 2021; Fernandez et al. 2019).

### 3.6 TCRβ Repertoire Features Enable Robust Prediction of Elevated PWV

**FIGURE 6:**
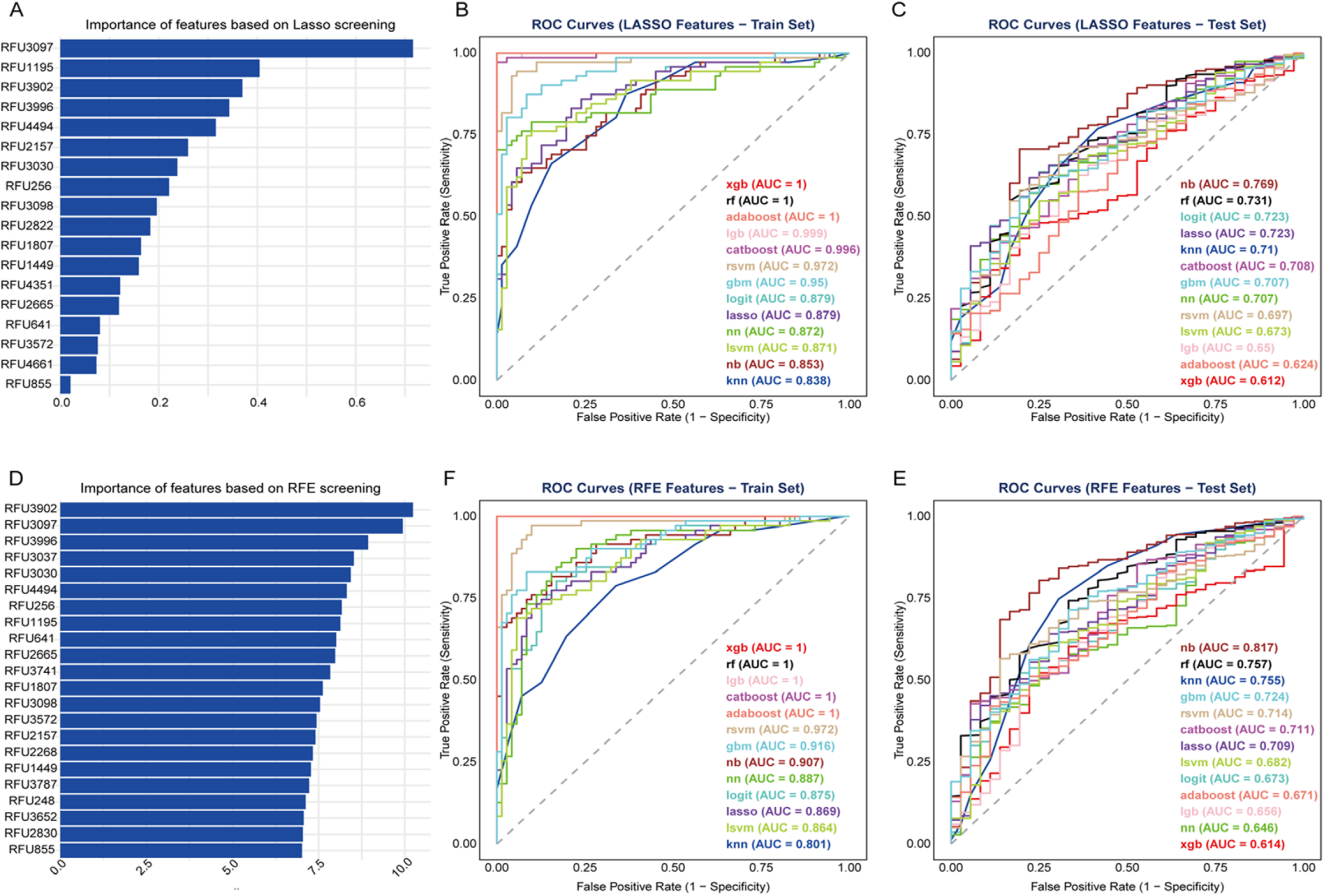
Machine learning‐based prediction of vascular stiffness using PWV‐associated TCRβ repertoire features. (A) Bar plot showing feature importance of PWV_related_RFUs selected by LASSO screening. (B) ROC curves showing model performance on the training set using LASSO‐selected features across 13 classifiers. (C) ROC curves on the test set using the same LASSO‐selected features. (D) Bar plot showing feature importance scores of PWV_related_RFUs selected via RFE. (E) ROC curves showing classifier performance on the training set using RFE‐selected features. (F) ROC curves on the test set using RFE‐selected features. Significance is evaluated by comparative AUC performance across models.

Given that vascular immune remodeling represents a distinct, CD4^+^ T cell‐driven signature of arterial stiffness, we finally assessed its potential as a non‐invasive biomarker. We trained multiple machine learning models using the 45 identified PWV_related_RFUs (Figure 4A). Feature selection was performed using both LASSO and RFE methods, retaining only RFUs with importance scores greater than zero (Figure 6A,D). A total of 17 and 22 RFUs were selected by LASSO and RFE, respectively.

We next constructed classifiers using 13 machine learning algorithms, including logistic regression, support vector machines, naïve Bayes, random forest, gradient boosting, deep neural networks, and others. Models were developed in the PSM‐matched development set and evaluated in the internal validation set (Figure 6B,C,E,F). While both methods yielded overlapping RFUs, RFE‐selected features performed better in downstream classification. Among all models, the naïve Bayes classifier using 22 RFUs selected via RFE achieved the highest performance, with an AUC of 0.907 in the PSM‐matched development set (Figure 6E) and 0.817 in the internal validation set (Figure 6F).

To further evaluate model performance in the presence of potential class imbalance, we generated a PR curve for this model. The naïve Bayes model achieved a PR‐AUC of 0.975 (Figure S5A), suggesting high discriminative power even in imbalanced clinical datasets. Collectively, these findings suggest that peripheral blood TCRβ repertoire features may serve as biomarkers for predicting arterial stiffness. The predictive power of repertoire‐based features further validates the biological relevance of vascular immune remodeling, suggesting these CD4^+^ T cell signatures are not only associative but mechanistically linked to early vascular dysfunction.

## 4 Discussion

In this study, we explored the immune mechanisms of vascular aging by associating peripheral TCRβ repertoire features with arterial stiffness. We identified a CD4^+^ T cell‐centered vascular immune remodeling process that appears distinct from classical immunosenescence and appears at least partially dissociable from canonical age‐related immune remodeling. Its unique signature is characterized by a paradoxical expansion of TCRβ repertoire diversity, driven by the broad, low‐frequency emergence of numerous functionally impaired CD4^+^ T cell clones—rather than the dominance of a few expanded clonotypes. This diffuse, subdominant clonal architecture is accompanied by heightened systemic inflammation.

Consistent with previous studies, our analysis confirmed that age, sex, and blood pressure are dominant independent determinants of PWV, underscoring their central role in driving arterial stiffness (AlGhatrif et al. 2013). Notably, after rigorous adjustment for these confounders through propensity score matching, individuals with elevated PWV exhibited significantly higher levels of systemic inflammation. This observation suggests that increased arterial stiffness is independently associated with an enhanced inflammatory state, even after adjustment for major physiological and metabolic covariates in adults without clinically overt cardiovascular disease. Previous studies have demonstrated that systemic inflammation can elevate arterial stiffness, either acutely or chronically, supporting the pro‐stiffening role of inflammation (Aminuddin et al. 2020; Vlachopoulos et al. 2005). Our findings, which reveal a persistent association between PWV and inflammation even after adjustment for major physiological covariates, reinforce the close interplay between vascular function and immune activation.

A key finding of this study is that elevated arterial stiffness was associated with increased TCR repertoire diversity. This pattern differs from Ma et al., who reported reduced TCRβ diversity and increased clonality in patients with essential hypertension and high baPWV (Ma et al. 2024). This difference may reflect distinct disease contexts rather than a simple contradiction. In our TORCH cohort of adults without clinically overt cardiovascular disease, the key comparisons were performed after adjustment for major physiological and metabolic covariates and further balancing by propensity score matching, and elevated baPWV was associated with increased Shannon diversity, reduced abundance of top expanded clones, and increased public clonotypes. By contrast, Ma et al. studied a disease‐defined hypertension cohort and described a more contracted, clonally skewed repertoire in patients with high baPWV. Together, these findings suggest that vascular immune remodeling may vary across clinical settings, with broader low‐frequency clonal recruitment in subclinical vascular stiffening and a more oligoclonal pattern in established hypertensive vascular disease.

At the repertoire level, our observations also differ from the contraction pattern typically seen in classical immunosenescence and autoimmune disease, which are commonly characterized by antigen‐driven oligoclonal expansion and reduced diversity (van de Sandt et al. 2023; Wang et al. 2025; Miao et al. 2023; Amoriello et al. 2021). In our study, the increased diversity appears to reflect broad, shallow remodeling across numerous low‐frequency T cell clonotypes rather than oligoclonal expansion of a limited number of dominant memory clones. This architecture is more consistent with diffuse repertoire perturbation than with classical antigen‐driven contraction or memory‐dominant clonal skewing. Supporting this interpretation, a substantial proportion of clonotypes enriched in individuals with high PWV were annotated to pathogen‐related immune responses, particularly involving viruses such as CMV and EBV. This observation is also consistent with epidemiological evidence from the MESA cohort showing that CMV infectious burden was the major correlate of Th1 bias and that Th1 bias was associated with subclinical atherosclerosis (Tracy et al. 2013). Together with the findings of Yu et al., who reported an independent association between PWV and CMV‐specific senescent CD8^+^ T cells, these observations support the possibility that chronic viral antigen exposure contributes to vascular immune remodeling (Yu et al. 2017). We also observed a significant reduction in the proportion of in‐frame TCRβ clonotypes in the PWV_High group, which may reflect altered repertoire maintenance and/or peripheral selection dynamics and provides further evidence of immune perturbation in individuals with elevated PWV.

A clearer distinction emerged when we examined the cellular origins of age‐ and PWV‐associated repertoire features. Our data show that repertoire alterations associated with chronological aging predominantly originate from CD8^+^ T cells, particularly MAIT subsets (Hu et al. 2024). In contrast, the PWV‐associated features of vascular immune remodeling are predominantly derived from CD4^+^ T cells. Although PWV‐related RFUs also increased with age, the lack of overlap between age‐associated and PWV‐associated RFUs supports the view that these represent dissociable T cell‐driven dynamics rather than a simple extension of classical chronological immunosenescence. This interpretation is also consistent with the published immune‐aging RFU study, which linked eRFUs primarily to highly differentiated CD8^+^ MAIT‐like cells and did not support a simple explanation by common viral exposure.

In this context, our findings extend prior human studies linking T‐cell aging to arterial stiffness. DeConne et al. showed that inflammatory and senescence‐related circulating T‐cell subsets, including CD4^+^ phenotypes, were associated with arterial stiffness in a large multi‐ethnic cohort (DeConne et al. 2025). Our data build on this observation by suggesting that, even in adults without clinically overt cardiovascular disease, vascular immune remodeling is characterized by a broader CD4^+^ T cell‐centered repertoire pattern rather than only shifts in selected circulating senescence‐related subsets. Transcriptional profiling provided a functional explanation for the paradoxical nature of this CD4^+^ T cell‐driven remodeling. PWV‐associated CD4^+^ T cells exhibited a gene expression program of chronic immune activation (e.g., Interferon‐γ response) coupled with proliferative arrest and metabolic impairment. These transcriptional changes mirror those implicated in the pathogenesis of atherosclerosis, highlighting a potential role for CD4^+^ T cell dysfunction in early vascular aging (Gaddis et al. 2021; Saigusa et al. 2022).

At a finer resolution, exact‐match mapping of PWVHigh‐enriched clonotypes to a paired single‐cell TCR reference atlas showed preferential representation within a naive‐like/early‐differentiation CD4^+^ compartment. This pattern raises the possibility that vascular immune remodeling may emerge, at least in part, at an earlier differentiation stage rather than being dominated exclusively by terminally differentiated or senescence‐associated effector subsets. Notably, this naive‐like mapping signal was accompanied by a lower in‐frame proportion in the PWVHigh group and by transcriptional features of chronic immune activation, impaired proliferative programs, and metabolic dysregulation in PWV‐associated CD4^+^ T cells. Together, these findings argue against a simple preservation of a healthy naive pool and instead support a dysfunctional CD4^+^ remodeling process that occupies a naive‐like/early‐differentiation neighborhood while exhibiting chronic activation, proliferative restraint, and metabolic impairment. Recent multi‐omic studies in healthy adults further indicate that early‐differentiation T‐cell states can undergo substantial transcriptional reprogramming before major compositional shifts become apparent, supporting the biological plausibility of an early‐state CD4^+^ remodeling process in our PWV‐associated analysis (Gong et al. 2025). Accordingly, the observed increase in TCR diversity is unlikely to reflect enhanced immune competence, but rather a broad remodeling of low‐frequency CD4^+^ clonotypes associated with chronic activation and proliferative restraint in adults with subclinical arterial stiffening.

Functionally, these transcriptional features suggest several mechanisms by which PWV‐associated CD4^+^ T cells may contribute to arterial stiffness, although the downstream pathways remain to be clarified. Prior studies have shown that activated CD4^+^ T cells, including Th1‐ and Th17‐associated populations, can promote arterial remodeling through pro‐inflammatory cytokines such as interferon‐γ and IL‐17, which affect vascular smooth muscle cells, extracellular matrix remodeling, and endothelial function (Eid et al. 2009). Chronic CD4^+^ T‐cell activation has also been implicated in oxidative stress and endothelial dysfunction through reduced nitric oxide bioavailability (Hausding et al. 2013). In this context, our transcriptomic data, particularly the enriched interferon‐response signature together with impaired proliferative and metabolic programs, support a chronically activated and dysfunctional CD4^+^ T‐cell state rather than definitive assignment to a single canonical helper subset. While these mechanisms have been most clearly characterized in advanced atherosclerosis and vascular disease, our findings suggest that related immune pathways may already be engaged in a broader and more subclinical phase of arterial stiffening.

Despite partial overlap with classical immunosenescence, particularly in features such as chronic antigenic stimulation and progressive T cell functional exhaustion, the immune remodeling observed in our study appears to follow a distinct immunological trajectory (Bektas et al. 2018; Singh et al. 2024; Mittelbrunn and Kroemer 2021). Classical immunosenescence is often driven by prolonged exposure to persistent antigens, particularly CMV, which induces large‐scale clonal expansions of terminally differentiated CD8^+^ T cells and contributes to significant TCR repertoire contraction (Pawelec et al. 2012; Sylwester et al. 2005; Vescovini et al. 2007). By contrast, the immune alterations observed in our study are marked by broader antigenic stimulation, likely involving multiple viral and microbial sources, and characterized by increased repertoire diversity and the accumulation of diverse low‐frequency clones, particularly within the CD4^+^ compartment. This immune remodeling pattern appears to differ from the dominant pattern of classical immune aging and may reflect broader, low‐intensity antigenic stimulation that contributes to vascular inflammation and stiffening.

Clinically, the distinct signature of vascular immune remodeling holds significant potential as a biomarker. Our findings demonstrate that immune repertoire features can reliably identify individuals with elevated PWV. Machine learning models trained on these features robustly classified arterial stiffness, extending the utility of TCR repertoire profiling—previously established in autoimmunity and cancer—to the emerging field of vascular aging (Yu et al. 2024; Miao et al. 2023). Furthermore, our findings open the possibility for immunomodulatory interventions targeting dysfunctional CD4^+^ T cell responses to attenuate vascular stiffening and delay cardiovascular aging.

The identification of CD4^+^ T cell dysfunction as a driver of vascular immune remodeling opens avenues for potential immunometabolic intervention. Notably, recent studies have demonstrated that NAD^+^ supplementation can restore the metabolic adaptability and effector function of exhausted or chronically stimulated CD4^+^ T cells across various disease contexts, including autoimmune disease (Tullius et al. 2014) and tumors (Wang et al. 2021). During vascular aging, a decline in NAD^+^ levels has been implicated in the development of arterial stiffness—a phenotype that can be ameliorated in preclinical models by NAD^+^ precursors such as nicotinamide mononucleotide (Abdellatif et al. 2021; Martens et al. 2018). Although direct evidence supporting an association between NAD^+^ supplementation and the reversal of CD4^+^ T cell dysfunction in vascular immune remodeling is currently limited, the transcriptomic profile of PWV‐associated CD4^+^ T cell clones—marked by proliferative arrest, chronic activation, and metabolic failure—mirrors hallmarks of NAD^+^ deficiency reported in aging and disease states (Chini et al. 2024; Covarrubias et al. 2021). These parallels suggest that NAD^+^‐boosting strategies could represent a targeted approach to mitigate early vascular immune remodeling, not merely by systemic anti‐aging effects, but by directly restoring the metabolic fitness of the specific dysfunctional CD4^+^ T cell populations we have identified. While still hypothetical, such interventions could offer a non‐invasive and scalable means of delaying arterial stiffening, particularly in the preclinical stages of vascular aging.

Nonetheless, several limitations should be acknowledged. First, as a cross‐sectional observational study, this work cannot determine the temporal order or causal direction linking CD4^+^ T cell remodeling and arterial stiffness. Although the observed immune features may represent an early process contributing to vascular stiffening, it is equally possible that vascular dysfunction and its associated inflammatory milieu secondarily reshape peripheral T cell states; thus, a bidirectional relationship cannot be excluded. Future longitudinal studies incorporating repeated baPWV measurements together with serial TCR repertoire profiling will be important to clarify the temporal sequence of these immune alterations relative to arterial stiffening. Mechanistic studies in vivo, as well as interventional studies targeting inflammatory pathways or vascular stiffness, will also be important to define causality more directly. Second, although major physiological and metabolic covariates were adjusted for in the regression analyses and balanced in the propensity score‐matched comparisons, some residual confounding cannot be excluded. In particular, medication use for hypertension or diabetes was not systematically collected in the present cohort and therefore could not be incorporated into the models. Smoking history was also not available in a form suitable for covariate adjustment. As both treatment status and tobacco exposure may influence arterial remodeling and immune phenotypes, future studies should incorporate detailed medication histories and smoking information to further refine confounder control. Third, our clonotype‐pathology annotations relied on curated public databases, which may be biased toward well‐characterized pathogen‐ or tumor‐associated epitopes and may not fully capture vascular‐relevant antigenic stimuli. The lack of serological data for chronic antigen exposures in the present cohort further precluded direct exposure‐stratified analyses. These constraints limit our ability to resolve the relative contribution of specific antigenic exposures to the observed repertoire remodeling. Direct validation using targeted approaches, such as peptide–MHC tetramer assays, cytokine profiling, antigen‐guided single‐cell analyses, or immunopeptidomics combined with TCR sequencing, will therefore be needed to improve antigen‐level specificity and clarify the contributions of defined CD4^+^ T cell subsets. Fourth, our analyses were restricted to peripheral blood, leaving uncertainty as to whether the immune remodeling observed in circulation is recapitulated within vascular tissues and the local microenvironment. Future studies integrating peripheral immune profiling with vascular tissue‐based analyses will be important to validate the tissue relevance of these circulating immune signatures and to define how CD4^+^ T cell dysfunction relates to local vascular remodeling.

In conclusion, our findings support a dual‐driver model of vascular aging: one trajectory linked to chronological age and a MAIT/CD8‐biased age‐associated immune remodeling signature, and another defined by CD4 T cell‐mediated vascular immune remodeling that does not simply mirror classical age‐related immunosenescence. This latter pathway features broad, persistent, low‐frequency clonal activation, reflecting widespread immune dysfunction that may accelerate arterial stiffening. By defining its immunological features and potential intervention targets, our work lays a foundation for precision immunology approaches to cardiovascular aging.

## Author Contributions

Y.M., T.L., B.C., and L.R. designed the research; Y.M. performed data analysis and data interpretation; Y.M. drafted the manuscript. All authors read and approved the final manuscript.

## Funding

This research was supported by the National Key Research and Development Program of China (No. 2020YFC2008002); The Science and Technology Research Project of Henan Province (No. 252102310056); Natural Science Foundation of Henan Province, China (No. 262300420181); Key Research and Development Program of Hubei Province (No. 2024BCB045). The Basic Research Fund of Henan Academy of Sciences (No. 240628018). The Startup Research Fund of Henan Academy of Sciences (No. 232016009).

## Ethics Statement

The studies involving human participants were reviewed and approved by the Ethical Clearance the Institutional Review Board of Tongji Hospital Tongji Medical College of HUST and BGI. All participants signed an informed consent form.

## Conflicts of Interest

The authors declare no conflicts of interest.

## Supporting information


**Figure S1:** Propensity score matching strategy and baseline covariate balance between PWV_High and PWV_Low groups.
**Figure S2:** Differences in V, J gene, VJ‐paired usage and CDR3 length distribution between PWV_High and PWV_Low groups.
**Figure S3:** Pathology‐associated clonotype annotation of PWV_High‐enriched TCR clonotypes.
**Figure S4:** Quantitative comparison of mapped cell‐type proportions for PWV‐associated clonotypes, Age‐related RFUs, and PWV‐related RFUs in the single‐cell reference atlas.
**Figure S5:** Precision‐Recall analysis of the naïve Bayes classifier for predicting arterial stiffness.

**Table S1:** Baseline physiological and clinical characteristics of the overall TORCH cohort.

**Table S2:** Clinical and physiological characteristics of the PSM‐matched development set.

**Table S3:** Differentially expressed genes of RFU^+^ T cells compared to their respective lineage counterparts based on single‐cell RNA‐seq analysis.

## Data Availability

The datasets supporting this study are available at Zenodo (accession: 15544872). The Supporting Information tables have also been deposited in Zenodo (accession: 17061482).
