# Supplementary material for: Vascular Immune Remodeling: A CD4 + T Cell–Driven Immune Trajectory Associated With Arterial Stiffness
Source: Aging Cell. 2026 Jun 4;25(6):e70568. doi: 10.1111/acel.70568 (PMC13580845; doi:10.1111/acel.70568)
Supplement: Supplementary file 1 [file ACEL-25-e70568-s002.docx]

## Supplementary Figure Legends

**Supplementary Figure 1 Propensity score matching strategy and baseline covariate balance between PWV_High and PWV_Low groups.**

(A) Bar plots showing age and gender distribution across the full cohort prior to matching. (B) Covariate balance plot showing standardized mean differences of selected physiological and biochemical variables before and after matching. Vertical lines indicate thresholds for acceptable balance. (C) Bar plots showing age and gender distribution in the matched PWV_High and PWV_Low groups. (D)Box plots show the difference in physiological and biochemical indicators between the matched PWV_High and PWV_Low groups, including age, body mass index (BMI), pulse, urine pH, systolic blood pressure (SBP), and diastolic blood pressure (DBP). All comparisons show no significant differences, confirming covariate balance. Significance is indicated as follows: ns: not significant.

**Supplementary Figure 2 Differences in V, J gene, VJ-paired usage and CDR3 length distribution between PWV_High and PWV_Low groups.**

(A) Boxplots showing covariate-adjusted comparisons of systemic inflammation level (SII), in-frame clone ratio, unique clone count, Shannon diversity index, and cumulative frequency of the top 100 clones. (B) Boxplots showing usage frequencies of individual V genes in the TCRβ repertoire across groups. (C) Boxplots showing usage frequencies of individual J genes in the TCRβ repertoire across groups. (D) Volcano plot displaying differential V–J gene pairing between groups. Red points indicate significantly enriched combinations in the PWV_High group and blue points indicate significantly enriched combinations in the PWV_Low group. (E) Boxplots showing the distribution of CDR3 amino acid lengths across groups. Wilcoxon test was used to compare length distributions between PWV_High and PWV_Low individuals. Significance is indicated as follows: *, P-value < 0.05; **, *P*-value < 0.01**;** *****, **P**-value **< 0.001**.

**Supplementary Figure 3 Pathology-associated clonotype annotation of PWV_High-enriched TCR clonotypes.**

(A) Histogram showing the distribution of CDR3 lengths for PWV-associated versus randomly sampled clonotypes. (B) Network graphs showing sequence similarity of PWV-associated clonotypes versus randomly sampled clonotypes. (C) UpSet plot showing the category-based annotation of 114 CDR3β sequences enriched in the PWV_High group, classified into pathogen-associated, autoimmune-associated, cancer-associated, and other clonotypes. ( D) UpSet plot showing the category-based annotation of 20 CDR3β sequences enriched in the PWV_Low group, classified into pathogen-associated, autoimmune-associated clonotypes. (E) UpSet plot showing the annotation distribution of 114 randomly selected TCRβ clonotypes from the overall repertoire, used as a background control. (F) UpSet plot showing the fine-grained pathogen annotation of PWV_High-associated CDR3β clonotypes mapped to microbial species, including various viruses and bacteria. Significance is indicated as follows: *, P-value < 0.05; **, *P*-value < 0.01**;** *****, **P**-value **< 0.001**.

**Supplementary Figure 4 Quantitative comparison of mapped cell-type proportions for PWV-associated clonotypes, Age-related RFUs, and PWV-related RFUs in the single-cell reference atlas.**

(A) Proportional distribution of reference cells matched to PWVHigh-enriched clonotypes across annotated CD4 T-cell subsets, based on exact CDR3 amino acid sequence matching in the single-cell reference atlas. PWVHigh-enriched clonotypes showed preferential representation in naive-like and early-differentiation CD4 compartments. (B) Quantitative comparison of the proportions of mapped reference cells across major immune cell compartments for Age-related RFU-associated cells. Age-related RFU-associated cells were predominantly represented in the MAIT compartment, with lower proportions in CD4, CD8, B-cell, myeloid, and NK compartments. (C) Quantitative comparison of the proportions of mapped reference cells across major immune cell compartments for PWV-related RFU-associated cells. PWV-related RFU-associated cells were predominantly represented in the CD4 compartment, with lower proportions in CD8, MAIT, and NK compartments. Statistical comparisons are indicated in the plots. Significance is indicated as follows: *, P-value < 0.05; **, *P*-value < 0.01**;** *****, **P**-value **< 0.001**.

**Supplementary Figure 5 Precision‐Recall analysis of the naïve Bayes classifier for predicting arterial stiffness**

(A) Precision–recall (PR) curve of the Naïve Bayes classifier trained on RFE-selected PWV_related_RFUs, evaluated on the independent test set. The area under the PR curve (PR-AUC) was 0.975, indicating high classification performance even in the presence of class imbalance. The dashed line represents the baseline precision (positive class prevalence = 0.914).
